# A Perspective on Natural and Nature-Inspired Small Molecules Targeting Phosphodiesterase 9 (PDE9): Chances and Challenges against Neurodegeneration

**DOI:** 10.3390/ph14010058

**Published:** 2021-01-13

**Authors:** Giovanni Ribaudo, Maurizio Memo, Alessandra Gianoncelli

**Affiliations:** Department of Molecular and Translational Medicine, University of Brescia, 25121 Brescia, Italy; maurizio.memo@unibs.it (M.M.); alessandra.gianoncelli@unibs.it (A.G.)

**Keywords:** PDE9, Alzheimer’s disease, natural compounds, cGMP, neurodegeneration, xanthines

## Abstract

As life expectancy increases, dementia affects a growing number of people worldwide. Besides current treatments, phosphodiesterase 9 (PDE9) represents an alternative target for developing innovative small molecules to contrast neurodegeneration. PDE inhibition promotes neurotransmitter release, amelioration of microvascular dysfunction, and neuronal plasticity. This review will provide an update on natural and nature-inspired PDE9 inhibitors, with a focus on the structural features of PDE9 that encourage the development of isoform-selective ligands. The expression in the brain, the presence within its structure of a peculiar accessory pocket, the asymmetry between the two subunits composing the protein dimer, and the selectivity towards chiral species make PDE9 a suitable target to develop specific inhibitors. Additionally, the world of natural compounds is an ideal source for identifying novel, possibly asymmetric, scaffolds, and xanthines, flavonoids, neolignans, and their derivatives are currently being studied. In this review, the available literature data were interpreted and clarified, from a structural point of view, taking advantage of molecular modeling: 3D structures of ligand-target complexes were retrieved, or built, and discussed.

## 1. Introduction

Dementia currently affects 44 million people worldwide, a number that is constantly growing in light of increasing life expectancy. The symptoms of Alzheimer’s disease (AD) consist of memory, attention, personality, intellect, and speech impairments [1]. From a biochemical point of view, amyloid plaques in the brain and degeneration of cholinergic innervation in the hippocampus and the cerebral cortex are reported to be involved in disease onset and progression [2]. Current therapeutic options include acetylcholinesterase (AChE) inhibitors (donepezil, rivastigmine, galantamine) and compounds, such as memantine, that target *N*-methyl-d-aspartate (NMDA) receptors. However, limited efficacy and frequently occurring adverse effects limit their use and prompt the research towards novel tools to contrast neurodegeneration [3].

In this context, phosphodiesterases (PDEs) are attracting growing attention as potential targets for small molecules as novel drug candidates [4,5]. PDEs hydrolyze the second messengers 3’,5’-cyclic adenosine monophosphate (cAMP) and 3’,5’-cyclic guanosine monophosphate (cGMP) with different degrees of selectivity, depending on the enzyme family. PDE4, PDE7, and PDE8 are in fact cAMP-selective, while PDE5, PDE6, and PDE9 are cGMP-selective. On the other hand, PDE1, PDE2, PDE3, PDE10, and PDE11 are “dual substrate” enzymes [6]. In general, PDEs are involved in intracellular signal transduction by regulating cAMP and/or cGMP levels.

PDE5 inhibitors acting on peripheral enzymes, such as sildenafil and its analogues, currently find application in clinic as therapeutic approaches against erectile dysfunction and pulmonary hypertension. Moreover, compounds targeting PDE5 are being studied for hearth failure, asthma, diabetes, obesity, and central nervous system (CNS) diseases [7,8,9]. The PDE4-selective inhibitor rolipram was studied as a potential treatment for AD, but its further development failed due to adverse effects [4,7].

Concerning neuroprotection, cognitive enhancing, and the effects against neurodegeneration, increased cAMP and cGMP levels by PDE inhibition are involved in promoting neurotransmitter release, amelioration of microvascular dysfunction and neuronal plasticity [10,11,12]. Stimulation of cAMP response element binding protein (CREB) and brain derived neurotropic factor (BDNF), together with improved release of nitric oxide (NO), are reported as the main biochemical mechanisms involved [4]. PDEs are expressed in several tissues. Except for PDE11 and for PDE6, which is only present in the pineal gland, all PDE families are expressed, to different extents, in the central nervous system [13]. Most importantly, several studies investigated the variations of PDE isoforms expression levels in the brain of AD patients: limited changes, mainly referred to PDE1, PDE4, PDE9, and PDE10, were noted [14,15,16,17].

PDE9 is among the currently most studied innovative targets for developing inhibitors to combat dementia [5,18], in light of its expression in the brain and of its unique structural features that allow the design of molecules selectively interacting with this target, as will be discussed in the following. PDE9 is endowed with high affinity for cGMP (K_m_ = 70 nM) and the highest selectivity over cAMP (K_m_ = 230 μM) among the other isoforms [19,20]. Moreover, it has been found to be highly expressed in brain of AD patients [4]. In particular, this isoform was detected in the amygdala, cerebellum, cortex, hippocampus, hypothalamus, midbrain, olfactory bulb, and striatum [21]. More recently, Patel and colleagues investigated the subcellular localization of PDE9 in the brain, highlighting changes during lifespan [22]. This enzyme is also expressed in the gut, kidney and heart [19]. For more general reviews on the inhibitors currently undergoing preclinical and clinical investigations that target PDE subtypes in brain, the reader is invited to consider our previous work and the paper by Prickaerts et al. [3,5].

The present contribution aims at providing an overview of the reports available in the literature concerning natural or nature-inspired PDE9 inhibitors, and a particular focus will be placed on the structural aspects of PDE9 that encourage the development of selective inhibitors, taking advantage of unique conformational features that allow further optimization of the scaffolds of natural compounds. Current review articles regard mainly PDE1–PDE5 inhibitors [23], while no updated overviews were retrieved concerning recent findings about other isoforms, and PDE9 in particular. 

For the preparation of this review, more than 60 contributions in the literature from the 2000–2020 timeframe were considered and screened. Original research articles were retrieved searching the PubMed (www.ncbi.nlm.nih.gov/pubmed/) and Scopus (www.scopus.com) databases using keywords such as “phosphodiesterase”, “PDE9”, “natural compounds”, “neurodegeneration”, “Alzheimer’s disease” and their combinations. The 3D models of the studied protein and complexes were retrieved from the Protein Data Bank (PDB, www.rcsb.org) and UCSF Chimera software was used to prepare the models [24]. All the 3D models included in this review are original and were produced for the current work.

## 2. Chemical Classes of Natural Compounds Targeting PDEs

The inhibition of phosphodiesterase activity was described for the first time at the end of the 19th century, when the natural alkaloid caffeine was reported to have bronchodilator properties. Some decades later, caffeine was identified as a nonselective PDE inhibitor [3]. Several plant extracts and, more specifically, their bioactive constituents were reported to possess neuroprotective action and to improve cognitive function against dementia and other neurodegenerative disorders [25]. The activity of natural compounds ameliorating AD conditions in preclinical or clinical studies is generally related to a multi-target mechanism of action [26], and inhibition of one or more PDE isoforms is often involved [8,27,28,29]. Natural, semi-synthetic, and synthetic compounds can in fact inhibit PDEs with different degrees of selectivity, as well as act as non-selective inhibitors [30].

It must be pointed out that the research field of PDE inhibition is traditionally connected with the world of natural compounds, as it was initially focused on alkaloids such as caffeine, as cited above, or theophylline and papaverine. However, it was later found that the effects of such compounds on blood vessels were mostly connected with the inhibition of cAMP-selective PDEs [23]. Within the broad class of alkaloids, chelerythrine and glaucine were also studied as natural PDE inhibitors but limited isoform selectivity and IC_50_ values in the higher μM range limited their further development [31].

Flavonoids are among the most widely studied classes of natural PDE inhibitors, even if the great majority of contributions in the literature are focused on the evaluation of such compounds against PDE5 [8,32,33,34,35]. Concerning this class, hesperidin and naringenin were reported to induce relaxing effects of noradrenaline-, okadaic acid-, and KCl-precontracted aorta. This activity is mediated by inhibition of cGMP hydrolysis by PDEs [36,37]. Dioclein and epigallocatechin-3-gallate (EGCG) were also studied as a vasorelaxant agents and PDE inhibitors interacting with the cGMP pathway, but the compound’s lack of selectivity was demonstrated to interact potently with PDE1 [38,39].

**Figure 1:**
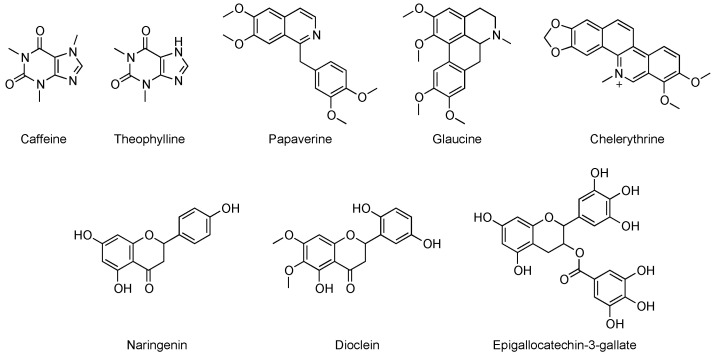
Chemical structures of some of the known natural phosphodiesterase (PDE) inhibitors.

Saponins, lignans, polyphenols, organic acids, and coumarins represent other examples of chemical classes of natural compounds known as non-specific PDE-inhibitors [8]. The chemical structures of the main cited compounds are reported in Figure 1.

## 3. Natural and Nature-Inspired PDE9 Inhibitors

The promising results of in vitro and in vivo studies, and the growing interest of major pharmaceutical companies, encourage medicinal chemists to explore the field of selective PDE9 inhibitors as a novel approach against dementia [18,40]. Synthetic PDE9 inhibitors are indeed currently being investigated in clinical trials as promising therapeutic options against neurodegeneration [41]. On the other hand, xanthines and polyphenols, as well as lignans, are among the most widely studied natural scaffolds for developing inhibitors interacting with PDEs in the CNS. Peculiar structural features of PDE9 isoform and chemical aspects of the investigated nature-inspired ligands will be discussed in the following sections.

### 3.1. Structural Features of PDE9

**Figure 2:**
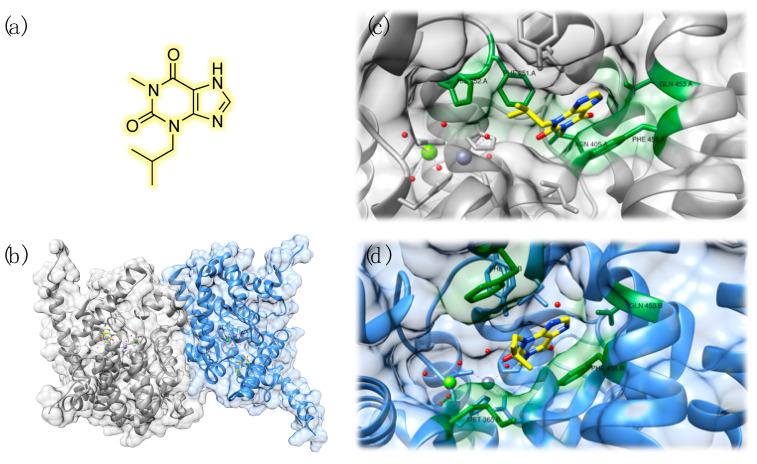
Chemical structure of 3-isobutyl-1-methylxanthine (IBMX) (**a**). Three-dimensional structure of PDE9 in complex with its inhibitor IBMX, highlighted in yellow (PDB ID: 2HD1): chain A is depicted in grey and chain B in blue (**b**). Detailed view of the interaction pattern of IBMX with chain A: zinc is represented in purple and magnesium is reported in green. Interacting residues have been labeled and highlighted in green (**c**). Detailed view of the interaction pattern of IBMX with chain B: zinc is represented in purple and magnesium is reported in green. Interacting residues have been labeled and highlighted in green (**d**).

The chemical structure of PDE inhibitors and their interaction pattern with the enzyme binding pocket have been extensively studied, and some pharmacophoric features of PDE ligands were highlighted [42]. Gln453 and Phe456 are the main residues involved in ligand–target interaction in the context of PDE inhibition and such amino acids are conserved within PDE families [43]. Huai et al. clearly resumed the structural features of PDE9, focusing on the study of the crystal structure of the PDEA2 isoform in complex with 3-isobutyl-1-methylxanthine (IBMX), a non-specific PDE binder (Figure 2). This compound, in fact, also interacts with PDE4, PDE5, and PDE9, inhibiting these enzymes in the μM range [20].

The catalytic domain of PDE9 is constituted by 16 helices (residues 181–506) and it is very similar to that of PDE4. On the other hand, a structural comparison with PDE5 highlighted significant differences [44]. Moreover, in crystal structures, PDE9 catalytic domains associate in dimers, while they further combine in tetramers in the case of PDE4 [20]. As well as in PDE4 and PDE5, zinc and magnesium are present inside PDE9, even if biochemical studies demonstrated that manganese also activates the protein [19]. Zinc forms coordination bonds with His256, His292, Asp293, Asp402, and two water molecules. The second ion is coordinated by Asp293 and five water molecules [20]. These details and the overall 3D structure of PDE9 are depicted in Figure 2.

Huang et al. reported a detailed study concerning the biorecognition of PDE9 by a single enantiomeric compound, in light of a peculiar structural asymmetry of the protein. In particular, the authors demonstrated the presence of a slight conformational difference between M-loops of PDE9 dimers and, most importantly, of a small hydrophobic pocket that may be targeted by enantiomerically pure compounds [45]. This feature will be discussed in detail in the following sections of the review. In this connection, it has also been observed that IBMX shows different orientations when interacting with other PDE isoforms. Nevertheless, as anticipated, key interactions, such as stacking between xanthine and phenylalanine and hydrogen bond between N_7_ and glutamine, are maintained. Interestingly, a slightly different binding motif for IBMX was also highlighted within the two monomers of the PDE9 dimer. These two ligands have a rotation difference of 50°: stacking is conserved but IBMX interacts with Phe251, His252, and Asn405 in one monomer and with Met365 and Phe441 in the other. Moreover, the isobutyl chain adopts different orientations (Figure 2) [20].

### 3.2. PDE9 Inhibitors Undergoing Clinical Studies

**Figure 3:**
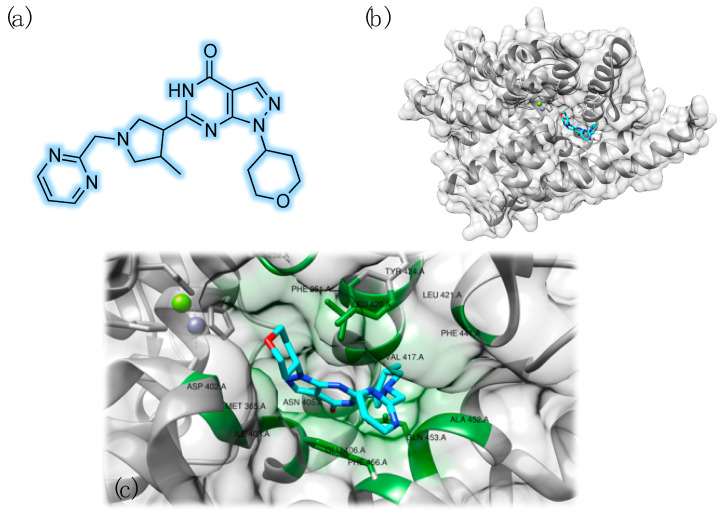
Chemical structure of the PDE9 inhibitor PF-04447943 (**a**). Three-dimensional structure of the complex with PDE9 (PDB ID: 4E90) (**b**). Detailed view of the interaction pattern of PF-04447943 with PDE9: zinc is represented in purple and magnesium is reported in green. Interacting residues have been labeled and highlighted in green (**c**).

The growing understanding of the individual physiological role and tissue localization of specific PDE isoforms paved the way for targeting more precisely cyclic nucleotide signaling pathways involved in specific diseases, such as dementia [46]. Several synthetic PDE9 inhibitors are under investigation in this context. Beneficial effects of PDE9 inhibitor BAY 736691 were reported in object recognition and passive avoidance tests in animal models, suggesting its memory enhancing role [47]. Li et al. studied the effects of this selective compound on in vitro and in vivo models of AD [48]. In this connection, it must be noted that Pfizer and Boehringer entered Phase II trials with PDE9-selective inhibitors, named PF-04447943 (Figure 3) and BI 409306, respectively [49,50].

**Figure 4:**
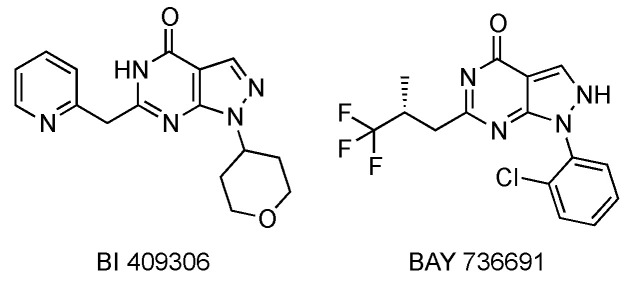
Chemical structures of the PDE9 inhibitors BI 409306 and BAY 736691.

Moschetti et al. reported the results of safety, tolerability, and bioavailability studies of BI 409306 on healthy human male subjects [50]. The chemical structures of this compound and of BAY 736691 are reported in Figure 4.

The compounds investigated to date to combat neurodegeneration showed moderate or at least partial specificity for PDE9, as these molecules are also substrates of PDE1 [44]. E2027 is another promising compound developed by Eisai: it is currently being examined in clinical trials as it provides neuroprotection in dementia by increasing central cGMP levels and improving neuronal plasticity [41,51]. Not many details have been provided so far, and even if some early reports in the literature classified this compound as a PDE1 inhibitor, it is currently referred to as a “selective PDE9 inhibitor” (IC_50_ = 3.5 nM, 1000-fold selectivity over other isoforms) [52,53]. In animal models, E2027 has been shown to inhibit the degradation of cGMP, increasing its levels in cerebrospinal fluid. Improved memory retainment and amelioration of cognitive impairment were among the observed effects [52]. A clinical study is investigating efficacy, safety and tolerability of E2027 in patients with dementia with Lewis bodies (ClinicalTrials.gov Identifier: NCT03467152).

### 3.3. Studies on Natural and Nature-Inspired Compounds Targeting PDE9

Several plant extracts were previously described to possess cGMP-specific PDE inhibitory activity, providing a hope for a potential application against cognitive disorders. Temkitthawon et al. reported a list of flavonoids endowed with this role [27], and other compounds, such as isoquiritigenin, icariin, anthocyanin, and sophoflavescenol, were reported afterwards [4].

**Figure 5:**
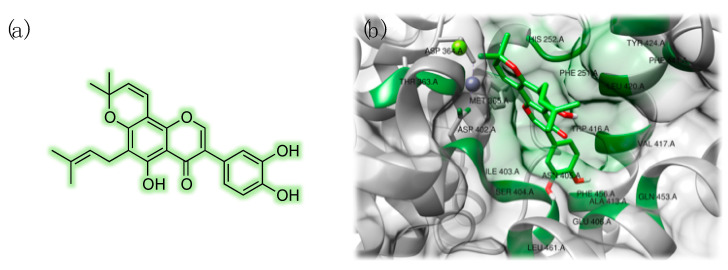
Chemical structure of pomiferin (**a**). Detailed 3D view of the predicted interaction motif with PDE9: zinc is represented in purple and magnesium is reported in green. Interacting residues have been labeled and highlighted in green (**b**). Interaction model was built based on the computational data reported by Ongaro et al. [54].

In a recent study, our research group adopted a set of in silico tools to screen a panel of chemically variegated natural compounds (flavonoids, polyphenols, organic acids, alkaloids, and anthocyanins) against PDE isoforms, including PDE9 (PDB ID: 3JSW) [54]. As a first step, compounds lacking drug-like pharmacokinetic features were excluded from the subsequent validated multi-docking screening. According to the results of this study, in terms of calculated binding energy (ΔG), flavonoids were highlighted as the most promising class of PDE9 inhibitors, with kraussianone 2, kraussianone 3, osajin, and pomiferin showing ΔG values < −11 kcal/mol. More specifically, pomiferin (ΔG = −11.6 kcal/mol) was predicted to be selective for PDE9, as weak interactions with other isoforms were computed. The predicted interaction pattern of pomiferin with PDE9 is reported in Figure 5.

**Figure 6:**
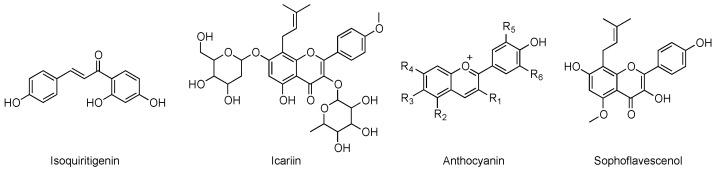
Chemical structures of some of the known c-GMP-specific PDE inhibitors.

Notably, in the same study, a lack of selectivity was highlighted for caffeine, that showed overall poor binding energies towards all the tested PDE isoforms (between −5.8 and −7.3 kcal/mol) [54]. The chemical structures of the natural compounds discussed in this section are reported in Figure 6.

**Figure 7:**
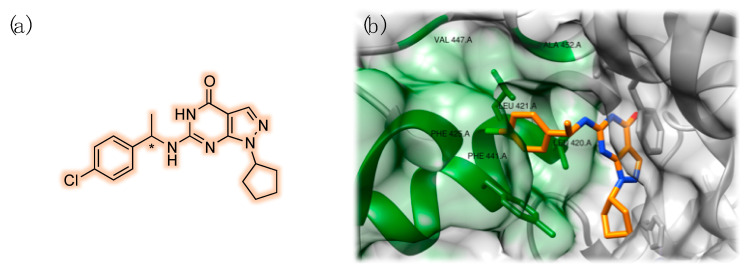
Chemical structure of (S)-C33/(R)-C33 (**a**). Detailed view of the interaction pattern of (S)-C33 with PDE9: the residues forming the specific hydrophobic M-pocket have been labeled and highlighted in green (PDB ID: 4Y8C) (**b**).

On the other hand, caffeine, xanthines and xanthine-inspired compounds were widely investigated as structures for developing PDE9 inhibitors. In particular, the pyrazolopyrimidinone scaffold appears particularly promising, and its chemical structure closely resembles that of naturally occurring xanthines [45,55]. In this connection, in their investigation for PDE9 inhibitors, Shao et al. reported a compound capable of targeting a small hydrophobic pocket in the protein structure. This peculiar arrangement of the molecule, named 3r, allowed 800-fold selectivity over PDE1 and 35,000-fold selectivity over PDE4 [56]. This specific pocket is constituted by residues Leu420, Leu421, Phe425, Ala452, and Phe441 (Figure 7). Most importantly, this region represents a unique structural feature of this isoform and, thus, it could be employed for designing compounds selectively targeting PDE9. Huang et al. further investigated this aspect and found that this site, named M-pocket, can be selectively target by a chiral compound, named (S)-C33 (Figure 7). This molecule showed improved potency and isoform selectivity when compared to its enantiomer (R)-C33, supporting the experimental hypothesis of the authors [45].

**Figure 8:**
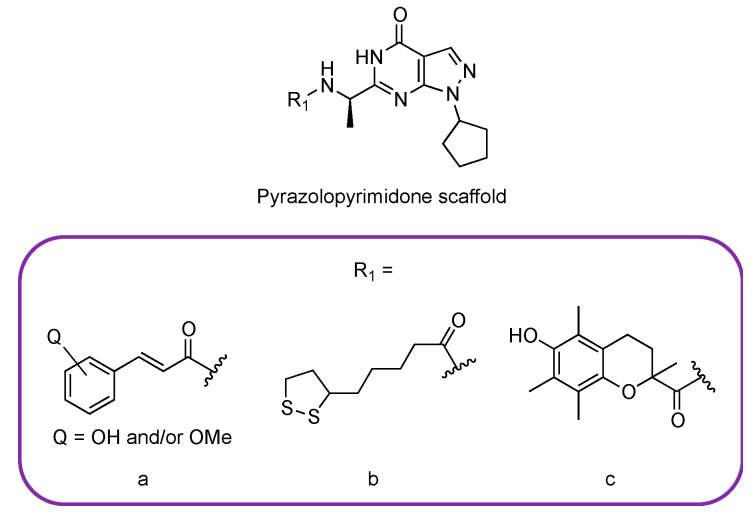
Chemical structures of the modified pyrazolopyrimidinone scaffold and of the moieties studied by Zhang et al., which are functional groups inspired by the structures of cinnamic acid (**a**), lipoic acid (**b**), and trolox (**c**) [57].

As stated above, most of the currently studied nature-inspired PDE9 inhibitors are based on the pyrazolopyrimidinone scaffold, and this also holds true for synthetic compounds undergoing clinical investigation [44]. Moreover, starting from this structure, Zhang et al. prepared a set of hybrid compounds generated from the combination of the pyrazolopyrimidinone scaffold with natural, antioxidant moieties (Figure 8). The authors studied the potential activity of such molecules against AD in vitro. These “multi-functional” compounds, combining PDE inhibitory activity and antioxidant properties, were designed with the aid of computational tools, such as molecular docking and molecular dynamics simulations. Most of the synthesized derivatives showed IC_50_ values < 200 nM on PDE9, with the derivative bearing the cinnamic acid group reaching IC_50_ = 56 nM. The best performing compound inhibited the enzyme with a potency comparable to that of the reference compound, BAY 736691. Interestingly, the most active derivatives also showed a good selectivity profile over PDE1 (between 25- and 40-fold) and PDE8 (between 250- and 400-fold). The authors investigated the antioxidant activity of the derivatives in the oxygen radical absorbance capacity assay (ORAC-FL), again highlighting the performance of the compounds bearing the cinnamic group as substituent in the scaffold. Eventually, the lack of toxicity on human neuroblastoma cells was assayed [57].

**Figure 9:**
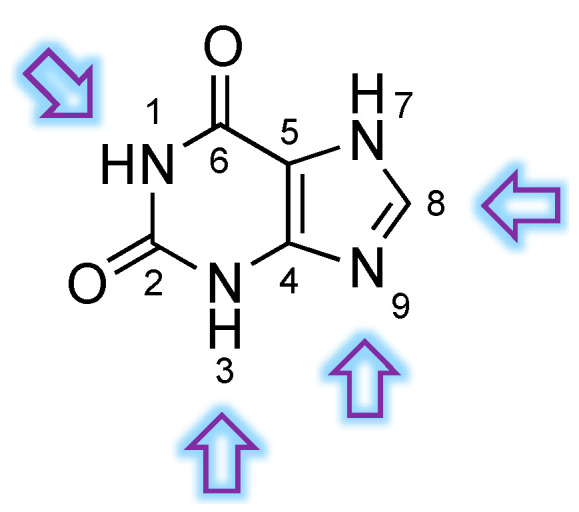
General structure of the xanthine scaffold. The positions that were modified in the abovementioned study have been marked with an arrow.

Singh et al. generated a library of 200 xanthine-based small molecules that were investigated for their selectivity as PDE9 ligands, with respect to other PDE isoforms. More specifically, the authors designed several groups of molecules that were modified at the N_1_, N_3_, N_9_, and C_8_ positions (Figure 9) [44].

Positions N_1_ and N_3_ were found to be not suitable for modifications: derivatization with aliphatic fragments induces loss of interaction with the PDE9 pocket. On the other hand, modification with aromatic fragments on the C_8_ position, along with derivatization on N_1_ and N_3_, allowed the generation of an extended scaffold capable of occupying the whole binding site more efficiently. With the aim of investigating isoform selectivity, the authors performed an in silico comparative interaction analysis with cAMP-specific PDE4, PDE7 and PDE8. Interestingly, bulky modifications on N_1_ and C_8_ induced a different orientation of the ligand when interacting with the catalytic subunit of other PDEs, thus suggesting a selective recognition of PDE9. Additionally, the side-activity on dual specific PDE1, PDE2, PDE3, and PDE10 was also investigated in the same study. As discussed above, cross-reactivity with PDE1 is detrimental for PDE9 inhibitors, especially in light of its high expression in the brain [50]. The modified xanthine-based compounds were predicted to be selective for PDE9, as less specific hydrophobic and van der Waals interactions were detected in the case of the residues of PDE1. Again, Tyr424 and Gln453 were confirmed to be crucial for selective binding [44].

**Figure 10:**
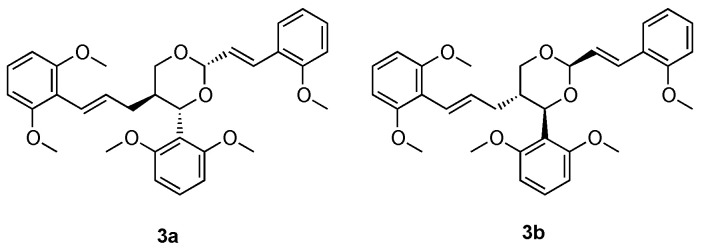
Chemical structures of the chiral (±)-torreyunlignans [58].

On the basis of the considerations about selective recognition of PDE9 by chiral compounds reported above, Cheng et al. investigated the activity of a class of (±)-torreyunlignans from *Torreya yunnanensis*. The authors reported the inhibitory activity of the ethanolic plant extract against PDE9, and the same effect was not observed on PDE4 and PDE5. (±)-Torreyunlignans represent a peculiar class of 8-9′-linked neolignans that were detected in these ethanolic extracts. The isolated compounds, in the form of single enantiomers, were tested against PDE9 and inhibited the enzyme in the μM range. The most promising compound, named **3a** in the paper, showed an IC_50_ value of 5.6 μM. Interestingly, the corresponding enantiomer **3b** showed similar activity (7.8 μM), suggesting that the chiral 1,3-dioxane moiety may have similar orientation within the PDE9 active site [58]. The chemical structure of the two compounds are reported in Figure 10.

**Figure 11:**
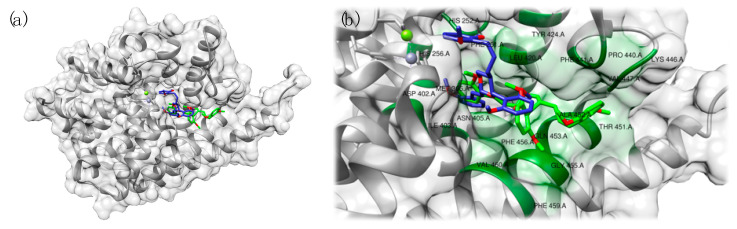
Results of the docking study based on the chemical structures of the natural PDE9 inhibitors (±)-torreyunlignans reported by Cheng et al. [58]. View of the full protein (**a**) and detailed view of the interaction pattern of **3a** (green) and **3b** (blue): the residues involved in the binding have been labeled and highlighted in green (**b**).

To better investigate the findings of the authors on these natural compounds interacting with PDE9 [58], we undertook a computational study to rationalize the experimental results and to evaluate the interaction pattern of the single enantiomers, according to the procedure briefly reported here. The protein model was retrieved from the PDB. Target and ligands were prepared for the blind docking experiment performed using Autodock Vina [59]. Receptor grid generation was automatically set, encompassing the whole protein (PDB ID: 2HD1). The number of docking poses was set to ten, and the other Autodock Vina parameters to default. Output data (energies and interaction patterns) were analyzed and scored using UCSF Chimera [24]. The values reported here are expressed in kcal/mol and refer to the most favored pose. The computed models are reported in Figure 11.

The docking study demonstrated, as expected, that both torreyunlignans interact with the catalytic subunit of PDE9. Calculated binding energy was found to be −9.0 kcal/mol for **3a** and −8.7 kcal/mol for **3b**, confirming the similar predicted activity observed by the authors in vitro, with **3a** being slightly more efficient [58]. As hypothesized by Cheng et al. [58], binding motif is similar for 1,3-dioxane but our study highlighted a different orientation of methoxyphenyl groups between the two compounds, and **3a** was predicted to bind key residues Gln453 and Phe456 in closer proximity (Figure 11).

## 4. Conclusions: The Perspective of the Medicinal Chemist

Natural compounds and plant extracts endowed with PDE inhibitory potential often also act through a combination of other biochemical mechanisms. In particular, plant-derived molecules showing PDE inhibitory activity that are promising candidates against neurodegeneration also have anti-amyloid and anti-tau effects, or, in other cases, are known to inhibit AChE [25]. This multi-target behavior has been reported for flavonoids [60] and for the components of *Ginkgo biloba*, *Poly tenuifolia*, and *Acorus calamus* extracts [61]. The presence of several mechanisms is perfectly in harmony with the growing interest towards the development of multi-target-directed ligands (MTDL) that take advantage of synergistic pharmacological effects [62]. Moreover, depending on their chemical structures, the neuroprotective effect of several natural and nature-inspired compounds, as outlined above, is also connected with their activity against reactive oxygen species (ROS) and inflammation [63,64,65,66]. In this connection, it was also reported that natural PDE inhibitors, by increasing cGMP levels, reduce the production of IL-6 and TNF-α [23]. On the other hand, it must also be considered that compounds showing promising performances in vitro and in preclinical models sometimes fail in clinical studies. In this connection, combined therapy could represent a solution: PDE9 inhibitors could enhance and support the effects of traditional therapy and pave the way for the development of more efficient therapeutic strategies.

It must be also pointed out that some of the synthetic compounds targeting PDE9 studied so far demonstrated detrimental off-target inhibitory activity on other isoform, resulting in undesired side effects [67]. Developing selective inhibitors to be applied in contrasting neurodegeneration requires considering the structural diversification highlighted in PDE9 [44], and this is made possible thanks to modern tools supporting drug discovery, such as molecular modeling. While PDEs share more than 75% identity when the catalytic domain is considered, PDE9 shows unique structural features. As demonstrated in this review, drug design can take advantage of such uniqueness to identify or build selective molecules on the basis of natural scaffolds. The presence of the peculiar hydrophobic M-pocket described above and of the asymmetry between the two subunits composing the dimer make PDE9 a suitable target to search for inhibitors with good selectivity. 

To date, xanthines, flavonoids, neolignans, and their derivatives have been studied as natural and nature-inspired compounds targeting PDE9. As chirality seems to play a crucial role in accessing the peculiar pocket identified in this isoform, and based on the structural asymmetry of PDE9, the world of natural compounds appears as an ideal source for identifying novel asymmetric scaffolds.

## Data Availability

Data sharing not applicable. No new data were created or analyzed in this study.
